# Medical Gas Plasma Jet Technology Targets Murine Melanoma in an Immunogenic Fashion

**DOI:** 10.1002/advs.201903438

**Published:** 2020-03-30

**Authors:** Sander Bekeschus, Ramona Clemen, Felix Nießner, Sanjeev Kumar Sagwal, Eric Freund, Anke Schmidt

**Affiliations:** ^1^ ZIK plasmatis Leibniz Institute for Plasma Science and Technology (INP Greifswald) Felix‐Hausdorff‐Str. 3 Greifswald 17489 Germany

**Keywords:** B16F10, kINPen, plasma medicine, reactive oxygen species, reactive nitrogen species

## Abstract

Medical technologies from physics are imperative in the diagnosis and therapy of many types of diseases. In 2013, a novel cold physical plasma treatment concept was accredited for clinical therapy. This gas plasma jet technology generates large amounts of different reactive oxygen and nitrogen species (ROS). Using a melanoma model, gas plasma technology is tested as a novel anticancer agent. Plasma technology derived ROS diminish tumor growth in vitro and in vivo. Varying the feed gas mixture modifies the composition of ROS. Conditions rich in atomic oxygen correlate with killing activity and elevate intratumoral immune‐infiltrates of CD8^+^ cytotoxic T‐cells and dendritic cells. T‐cells from secondary lymphoid organs of these mice stimulated with B16 melanoma cells ex vivo show higher activation levels as well. This correlates with immunogenic cancer cell death and higher calreticulin and heat‐shock protein 90 expressions induced by gas plasma treatment in melanoma cells. To test the immunogenicity of gas plasma treated melanoma cells, 50% of mice vaccinated with these cells are protected from tumor growth compared to 1/6 and 5/6 mice negative control (mitomycin C) and positive control (mitoxantrone), respectively. Gas plasma jet technology is concluded to provide immunoprotection against malignant melanoma both in vitro and in vivo.

## Graphical abstract

Cancer is among the leading causes of death worldwide, motivating the need for novel therapeutic concepts in oncology. In chorus, the immune system is a significant contributor to tumor control. Utilizing a novel medical gas plasma technology concept and melanoma as model tumor, reduction in tumor mass as well as antitumor immuno‐stimulation is demonstrated in a reactive oxygen species dependent fashion.

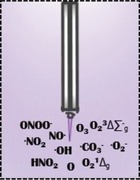

## 1 Introduction

Medical technologies from physics are irreplaceable for both diagnosis and therapy of many types of diseases. For instance, ionizing radiation still is the first‐line treatment in several types of cancers.^[^ ^1^ ^]^ Similarly, the concept of photodynamic therapy that is based mainly on the local production of singlet delta oxygen is used for the treatment of several malignant disorders.^[^ ^2^ ^]^ In 2013, a novel physics‐based therapy was added to the array of accredited therapies based on physics: medical plasma technology.^[^ ^3^ ^]^ This technology mainly acts via deposition of a plethora of different reactive oxygen and nitrogen species (ROS) deposited locally into the target tissue^[^ ^4^ ^]^ without causing thermal damage.^[^ ^5^ ^]^ The current indication of medical plasma therapy is to promote beneficial effects on the healing of chronic wounds and ulcers, apart from other dermatological indications.^[^ ^6^ ^]^ Strikingly, antitumor efficacy in head and neck cancer patients suggested plasma treatment to have a role in oncology as well.^[^ ^7^ ^]^ The concept linking these seemingly unrelated effects of stimulation of healing in non‐healing wounds on the one hand, and killing of tumor cells on the other hand is termed hormesis. ROS are a prime class of hormetically acting molecules that are stimulating molecules in intracellular signaling at low concentrations while having cytotoxic effects at higher concentrations.^[^ ^8^ ^]^ However, the species that are produced via gas plasmas are not necessarily the species that directly act on cells on tissues directly.^[^ ^4^ ^]^ Instead, secondary products and ROS derived from the primary ROS generated by plasmas are more likely to be the biological effectors^[^ ^8^ ^]^ that may also have immunological consequences.^[^ ^9^ ^]^

It is known not only since the Nobel Prize for Medicine or Physiology awarded in 2018 to checkpoint immunotherapy that the immune system plays a pivotal role in antitumor responses in patients.^[^ ^10^ ^]^ In particular, T‐cells are critical in selectively targeting malignant over non‐malignant cells.^[^ ^11^ ^]^ A prerequisite of antitumor T‐cell immunity is the availability of tumor antigens as well as a pro‐immunogenic context in which these antigens are displayed. Already, a decade ago, Obeid and colleagues discovered calreticulin (CRT) exposure to be vital in dictating the immunogenicity of tumor cell death.^[^ ^12^ ^]^ Accordingly, the recently reviewed paradigm of immunogenic cancer cell death (ICD) postulates that not only the event of cell death but also its inflammatory context is decisive for the immune system responding to dying tumor cells in a tolerogenic or immunogenic fashion.^[^ ^13^ ^]^

The first tumor entity showing the importance of anticancer immunity and checkpoint therapy is malignant melanoma.^[^ ^14^ ^]^ Melanoma is particularly stimulating to the immune system due to its extraordinary high mutation rate, which leads to the formation of several cancer‐neoantigens.^[^ ^15^ ^]^ Melanoma is therefore considered as model tumor when investigating novel therapeutic concepts linked to anticancer immunity.^[^ ^16^ ^]^ To this end, we investigated and confirmed both the efficacy and the immunogenicity of medical gas plasma treatment in an experimental model of syngeneic melanoma. To provide a measure of the degree of immunogenicity of the gas plasma treatment, we used a poorly immunogenic drug (mitomycin C) and a highly immunogenic drug (mitoxantrone) as reference treatments as outlined in a previous study.^[^ ^12^ ^]^

## 2 Results

### 2.1 Plasma Jet Treatment Oxidized and Killed Melanoma Cells by Gas Phase Derived ROS

**Figure 1:**
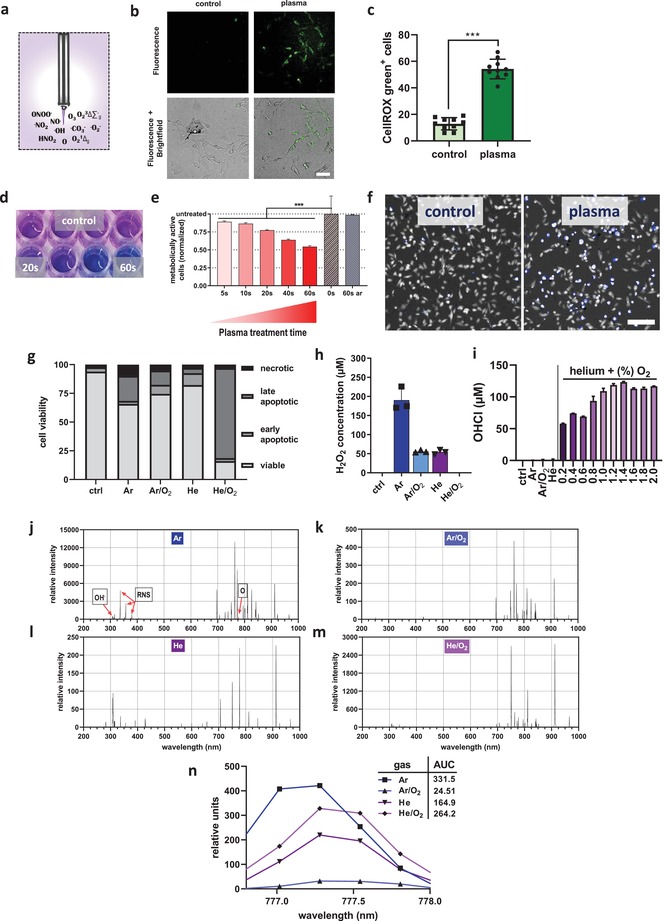
Plasma jet treatment oxidized and killed melanoma cells by gas phase‐derived ROS. a) Scheme of the medical plasma jet technology generating a multitude of ROS simultaneously. b) Representative brightfield and DCF fluorescence images of control and plasma‐treated B16F10 melanoma cells as well as c) quantification of fluorescence. d) Representative image of resazurin to resorufin turnover of cells in microplates and e) quantification of metabolic activity of melanoma cells exposed to plasma or left untreated (60 s ar = 60 s argon gas treatment alone with plasma off). f) Representative overlay images of digital phase contrast (white) and DAPI (blue) in control and plasma‐treated cells and g) flow cytometric viability analysis for each plasma gas setup. h,i) Quantification of H_2_O_2_ (h) and HOCl (i) in liquid with argon (Ar), argon/oxygen (Ar/O_2_), helium (He), and helium/oxygen (He/O_2_) plasma treatment. Optical emission spectroscopy (OES) spectra of j) argon (Ar), k) argon/oxygen (Ar/O_2_), l) helium (He), and m) helium/oxygen (He/O_2_) plasmas. n) Comparison of intensities at about 777 nm indicative for atomic oxygen and area under the curve (AUC) calculation (inlet). Data are mean ± SEM. Statistical comparison was performed using *t*‐test (c) and ANOVA against control cells (e). Scale bar is 20 µm (b) and 100 µm (f).

Medical gas plasma jet technology generates different types of ROS simultaneously (**Figure** **1**a). These ROS were capable of oxidizing murine B16F10 melanoma cells (Figure 1b) to a significant extent when compared to that of untreated cells (Figure 1c). Analyzing the metabolic activity of gas plasma jet treated melanoma cells (Figure 1d), a treatment time dependent decrease was observed that differed significantly from that of the untreated cells (Figure 1e). This decline was associated with terminal cell death (Figure 1f). The feed gas composition of a plasma jet determines its mixture of ROS in the plasma gas phase. Utilizing four different feed gas composition to ignite the medical gas plasma jet, namely, argon (Ar), argon/oxygen (Ar/O_2_), helium (He), and helium/oxygen (He/O_2_), a differential impact of each feed gas composition on the viability of plasma‐treated melanomas was observed (Figure 1g). An indirect measure of analyzing the reactive species composition of the plasma gas phase is determining the generation of long‐lived end products in the liquids exposed to plasma. While Ar plasma generates significant amounts of hydrogen peroxide (H_2_O_2_) in the liquid phase, primarily via hydroxyl radical (HO·) production, the He/O_2_ but not the He or Ar plasma setting was capable of producing hypochlorous acid (HOCl) in liquids (Figure 1i). Vice versa, the He/O_2_ condition did not generate H_2_O_2_ while the Ar condition did not generate HOCl. The Ar/O_2_ and He condition produced some H_2_O_2_ but not HOCl. The latter was dependent on the amount of O_2_ added to the He and was close to maximum at 1%. A direct measure of analyzing some types of ROS in the plasma gas phase is using optical emission spectroscopy. This technique captures the unique emission spectra of discharge plasmas with characteristic emission spectral lines for different types of atoms or molecules. For instance, the Ar plasma generates a visible peak for OH· at 307 nm and the second positive system of nitrogen for the bands immediately right of that line (Figure 1j). The bands above 700 nm mostly relate to Ar‐derived species and atomic oxygen (O) at 777 nm. Comparing the area under the curve of O for Ar (Figure 1j), Ar/O_2_ (Figure 1k), He (Figure 1l), and He/O_2_ (Figure 1m), O was present mainly in the Ar and He/O_2_ setups (Figure 1n). O_2_ addition was 1%. Altogether, plasma treatment oxidized and subsequently inactivated murine melanoma cells, and the degree of this inactivation was dependent on the feed gas composition and its resulting ROS mixture in the plasma gas phase.

### 2.2 Plasma Treatment of Syngeneic Melanoma Reduced Tumor Mass and Increased Leukocyte Tumor Infiltrates In Vivo

**Figure 2:**
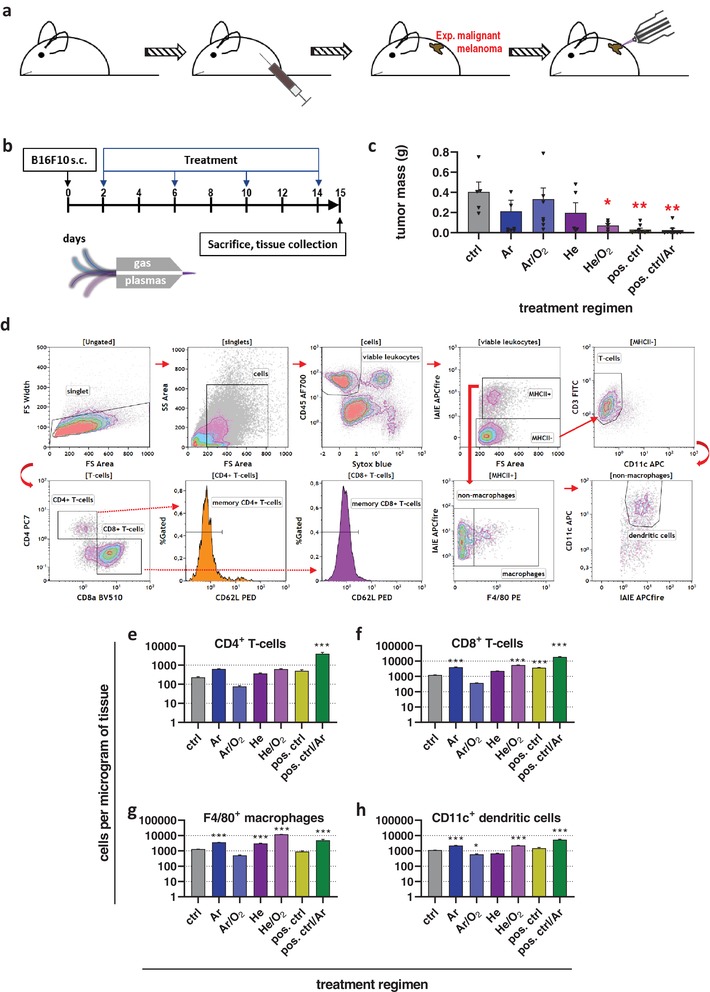
Plasma treatment of syngeneic melanoma reduced tumor mass and increased leukocyte tumor infiltrates in vivo. a) Workflow of in vivo experiment and b) treatment schedule; c) tumor mass of control and treatment groups; d) flow cytometry gating strategy to determine intratumoral leukocyte subpopulations; e–h) quantification intratumoral of CD4^+^ T‐helper cells (e), CD8^+^ cytotoxic T‐cells (f), F4/80^+^ macrophages (g), and CD11c^+^ dendritic cells (h) per microgram of tumor tissue. Data are mean ± SEM from two independent experiments. Statistical comparison was performed using ANOVA against the control group.

To investigate the antitumor efficacy of the four different gas plasma setups, C57BL/6 mice were inoculated with 1 × 10^5^ B16F10 melanoma cells on the left flank (**Figure** **2**a). Treatment of the tumor with gas plasma or the positive control (pos. ctrl; imiquimod) was performed four times before the sacrifice of animals and tissue collection (Figure 2b). Analysis of the tumor weight showed a reduction of tumor growth, which was significant for He/O_2_, positive control, and positive control plus Ar plasma (Figure 2c). After sacrifice, tumors were collected and digested using GentleMacs technology to retrieve viable, single‐cell tumor suspensions. Cell suspensions were labeled with fluorescently conjugated monoclonal antibodies targeting several immune cell subsets of the tumor microenvironment prior to performing multicolor flow cytometry. Leukocyte quantification was done by gating on single cells and the viable (Sytox Blue‐negative) leukocyte (CD45^+^) fraction among them (Figure 2d). The major histocompatibility class II (MHCII) negative cells were mostly T‐cells (CD3^+^), with the majority being cytotoxic CD8^+^ over helper CD4^+^ T‐cells. MHCII^+^ myeloid cells were gated for F4/80^+^ macrophages and CD11c^+^ dendritic cells (DCs). In our melanoma model, the majority of intratumoral T‐cells were of a memory (CD62L^−^) phenotype, which is consistent with findings in patients.^[^ ^17^ ^]^ This is because CD62L^+^ T‐cells preferentially home to secondary lymphoid organs that are lined with high endothelial venues, while CD62L^−^ T‐cells primarily prime into tissues such as tumors, where they patrol in search for their cognate antigen.^[^ ^18^ ^]^ Quantification of different intratumoral leukocyte subsets revealed a non‐significant increase of CD4^+^ T‐cells in all groups but Ar/O_2_, with the number of CD4^+^ cells in the positive control plus Ar plasma differing significantly from that in the untreated control tumors (Figure 2e). By contrast, CD8^+^ cytotoxic T‐cells were significantly increased in the groups showing the best tumor control (Figure 2c), namely Ar, He/O_2_, positive control, and positive control plus argon plasma (Figure 2f). A similar trend was observed for intratumoral macrophages, with the exception of a significant increase of macrophages in the He but not the positive control group tumors (Figure 2g). DCs, the prime cell type launching antitumor T‐cell responses, were significantly elevated in the Ar, He/O_2_, and positive control plus Ar plasma group, while in the Ar/O_2_ condition, they were significantly decreased (Figure 2h). In summary, medical gas plasma jet treatment reduced melanoma burden in vivo and stimulated intratumoral leukocyte infiltration, with the feed gas setting being decisive for both antitumor efficacy and immune infiltration.

### 2.3 Plasma Treatment of Murine Syngeneic Melanomas Increased T‐Cell Activation

**Figure 3:**
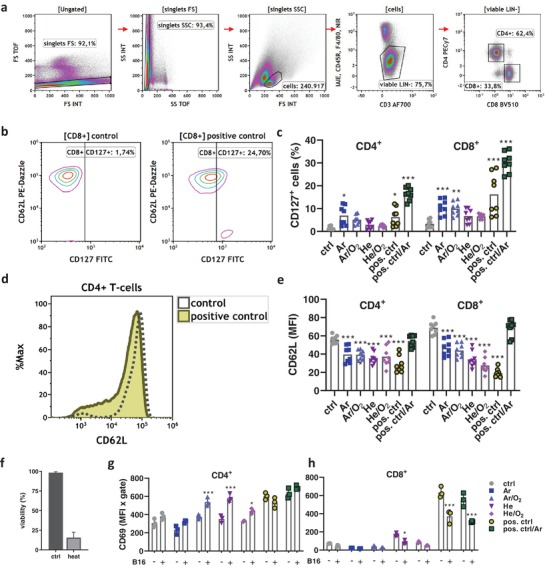
Plasma treatment of murine syngeneic melanomas increased T‐cell activation. a) Flow cytometry gating strategy to investigate CD4^+^ and CD8^+^ T‐cells from secondary lymphoid organs of tumor‐bearing mice; b) representative contour plots for CD62L and CD127 in CD8^+^ T‐cells from control and positive control animals; c) quantification of CD127 in CD4^+^ and CD8^+^ T‐cells from lymph nodes and splenocytes of tumor‐bearing mice; d) representative overlay histogram of CD62L expression on CD4^+^ T‐cells from control and positive control of tumor‐bearing mice; e) quantification of CD62L in CD4^+^ and CD8^+^ T‐cells from lymph nodes and splenocytes; f) viability of B16F10 cells in control conditions or after heat inactivation (65 °C for 3 min) as determined by flow cytometry; g,h) quantification of CD69 expression on CD4^+^ (f) and CD8^+^ (g) T‐cells from splenocytes of tumor‐bearing mice in presence or absence of B16F10 melanoma cells for 18 h. Data are mean ± SEM from secondary lymphoid organs extracted from animal experiments shown in Figure 2. Statistical comparison was performed using ANOVA against control (ctrl) group (c,e) or multiple *t*‐tests of cells in the presence (+) and absence (−) of melanoma cells (g,h).

Plasma‐treated tumors showed an enhanced immuno‐infiltration, and the next question was to assess activation levels of T‐cells, a subset of leukocytes known to mediate antitumor immune responses. Secondary lymphoid organs (lymph nodes, spleens) were collected, digested using GentleMacs technology, and viable CD4^+^ and CD8^+^ T‐cells were analyzed by multicolor flow cytometry (**Figure** **3**a). CD127, the interleukin (IL) 7 receptor, is a marker for T‐cell activation and differentiation. This marker was enhanced in T‐cells from lymph nodes of the animals receiving imiquimod antitumor therapy (Figure 3b). The quantification revealed a significant upregulation of CD127 in both CD4^+^ and CD8^+^ T‐cells in the Ar plasma, positive control, and positive control plus Ar plasma groups when compared to untreated controls (Figure 3c). Notably, all types of treatment increased CD127 expression. At the same time, a decrease of CD62L expression of T‐cells (Figure 3d) was observed in all mice receiving plasma or imiquimod anti‐melanoma therapy. A decrease of CD62L is known to be associated with T‐cell activation, and a significant decrease was observed in both CD4^+^ and CD8^+^ T‐cells in all groups except positive control plus Ar plasma (Figure 3e). It is unclear why the latter group receiving combination therapy did not show any decline in CD62L while the monotherapy did. To investigate whether T‐cells of tumor‐bearing mice were responsive to B16F10‐derived tumor antigen, splenocytes of tumor‐bearing mice were co‐cultured ex vivo with partially heat‐inactivated melanoma cells to free up antigen while at the same allowing the live‐cell fraction (Figure 3f) to provide for T‐cell stimulation. Analyzing the expression of the early activation marker of T‐cells, CD69, a significant increase was observed for the Ar/O_2_, He, and He/O_2_ conditions when comparing splenic CD4^+^ T‐cells cultured in the presence or absence of melanoma cells (Figure 3g). For CD8^+^ T‐cells, a significant decrease was observed in the He plasma and positive control groups (Figure 3h). Taken together, T‐cells of secondary lymphoid organs showed a higher baseline activation with therapy groups compared to controls, while the re‐stimulation of splenocytes with tumor cells ex vivo only partially led to enhanced T‐cell activation.

### 2.4 Gas Plasma Treatment Induced Immunogenic Cancer Cell Death in Melanoma Cells

**Figure 4:**
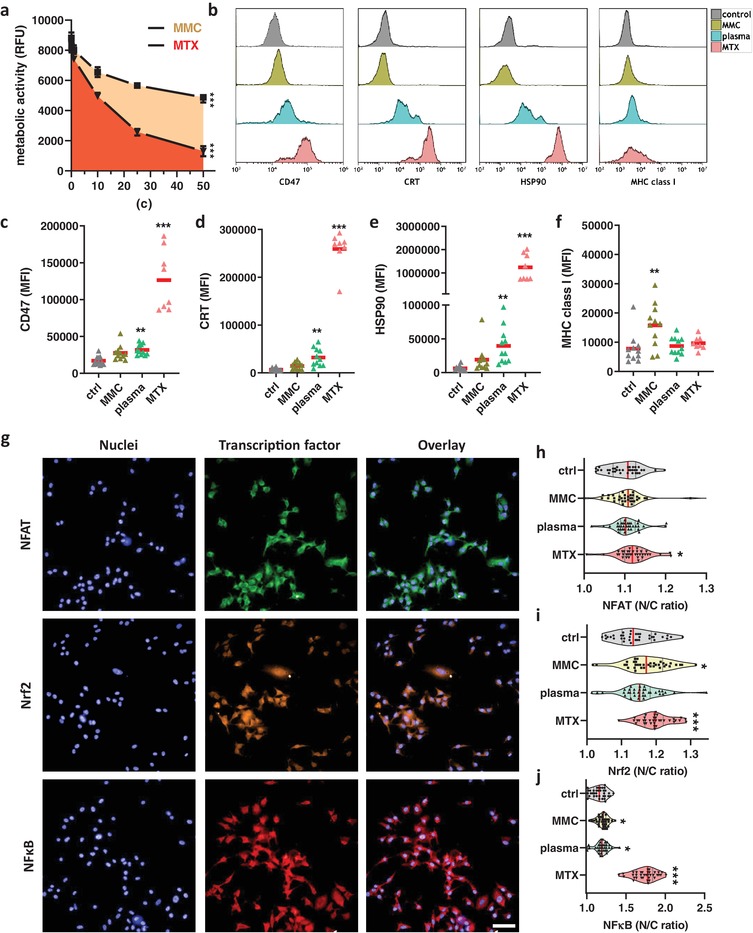
Argon gas plasma treatment induced immunogenic cancer cell death in melanoma cells. a) Metabolic activity of B16F10 melanoma cells incubated with varying concentrations of mitomycin C (MMC) or mitoxantrone (MTX) at 24 h; b) representative overlay histograms of expression intensities of CD47, calreticulin (CRT), heat‐shock protein 90 (HSP90), and major histocompatibility complex I (MHCI) on B16F10 melanoma cells at 24 h; c–f) quantitative comparison of mean fluorescent intensity (MFI) of cells at 24 h for CD47 (c), CRT (d), HSP90 (e), and MHCI (f); g) representative images of one field of view of nuclei (DAPI) and transcription factors (NFAT, Nrf2, NFκB) labeled with fluorescent antibodies as well as overlays in B16F10 melanoma cells; h–j) quantification of the nuclear to cytoplasmic (N/C) fluorescence intensity ratio for NFAT (h), Nrf2 (i), and NFκB (j), each dot represents data from four fields of view. Data from three experiments show mean ± SEM (a), mean (c–f), and violin plots and mean (h–j). Statistical analysis was performed using ANOVA against control cells. Scale bar is 50 µm. RFU = Relative fluorescence units.

Medical gas plasma jet treatment of syngeneic murine melanomas led to an increased immuno‐infiltration and an enhanced T‐cell activation profile. To analyze the immunogenic nature of gas plasma treatment of tumor cells, melanoma cells were exposed in vitro to argon gas plasma treatment or drugs known to have a low (mitomycin C, MMC) or high (mitoxantrone) immunogenic profile.^[^ ^12^ ^]^ The toxic action of the drugs was confirmed by the assessment of the metabolic activity melanoma cells (**Figure** **4**a). To investigate the ICD‐nature of the drugs and plasma treatment, multicolor flow cytometry was performed after 24 h of incubation to quantify the levels of the anti‐phagocytic molecule CD47, the eat‐me signal calreticulin (CRT), the ICD marker heat‐shock protein 90 (HSP90), and MHCI (Figure 4b). CD47 was significantly enhanced with plasma and MTX treatment (Figure 4c), suggesting a putative decrease of phagocytosis. At the same time, however, the pro‐phagocytic and immunogenic markers CRT (Figure 4d) and HSP90 (Figure 4e) were significantly increased with both the plasma and MTX treatment. MHCI, on the other hand, was significantly increased only with MMC treatment (Figure 4f). To analyze the transcription factors involved with the plasma and drug exposure, quantitative high content image analysis was performed for analyzing the nuclear translocation of the nuclear factor of activated T‐cells (NFAT), nuclear factor E2‐related factor 2 (Nrf2), and nuclear factor “kappa‐light‐chain‐enhancer” of activated B‐cells (NFκB). This was done by segmenting the nuclear (DAPI^+^) and cytosolic (DAPI^−^ and digital phase contrast^+^) region of each individual cell of an image. The nuclear over the cytosolic mean fluorescent intensity was calculated for each of the transcription factors as an indicator of their nuclear translocation and activation of downstream genes (Figure 4g). For each condition, about 20 000 individual cells were analyzed. For NFAT, algorithm‐driven quantification revealed a modest but significant increase for MTX but not for plasma and MMC (Figure 4h). For Nrf2, a significant increase was found with MTX and MMC (Figure 4i). MTX also facilitated a substantial and significant increase of NFκB, while that of MMC and plasma treatment was lower but still significantly enhanced compared to that of the untreated control cells (Figure 4j). In sum, plasma treatment increased immunogenic cancer cell death in melanoma cells, which was concomitant with elevated nuclear translocation of NFκB.

### 2.5 Vaccination with Plasma‐Treated Cells Protected from Melanoma Growth

**Figure 5:**
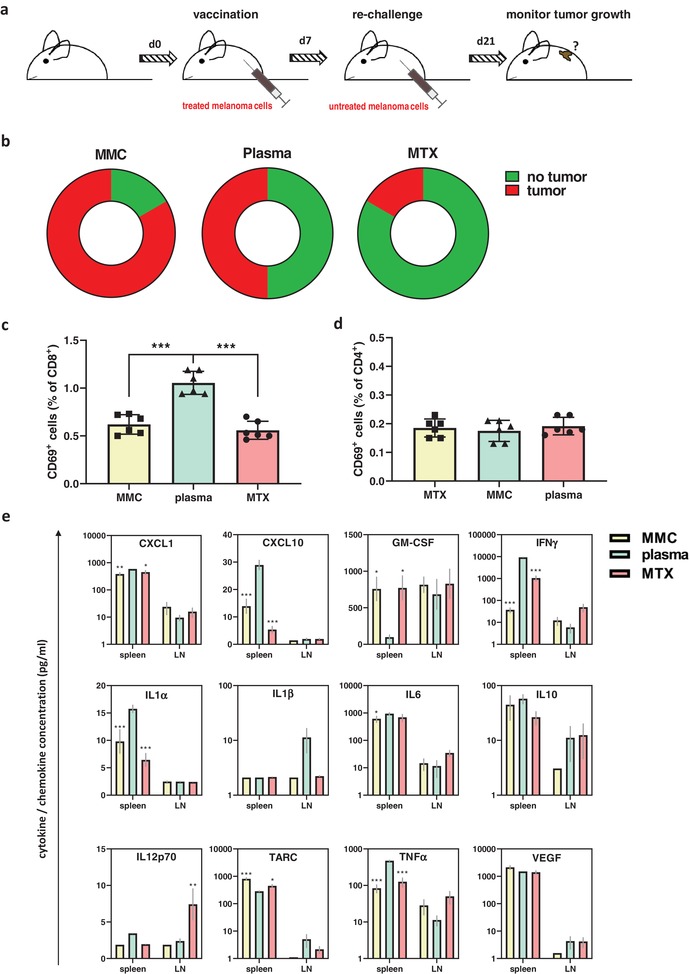
Vaccination with plasma‐treated cells protected from melanoma growth. a) Workflow of the vaccination animal experiment; b) quantification of the fraction of tumor‐bearing animals for each of the groups receiving cells exposed to either mitomycin C (MMC), plasma, or mitoxantrone (MTX)‐treated B16F10 melanoma cells prior to re‐challenge with viable cells; c,d) quantification of CD69 expression in CD8^+^ (c) and CD4^+^ (d) splenic T‐cells from vaccinated animals cultured with melanoma cells in vitro at 24 h; e) 12‐plex quantification of cytokines and chemokines in supernatant retrieved of splenocytes (spleen) or lymph node‐derived cells (LN) from vaccinated animals cultured with B16F10 melanoma cells at 24 h. Data are mean ± SEM from six animals per group. Statistical comparison was performed using ANOVA against the plasma group.

To investigate the in vivo relevance of plasma‐induced ICD identified in vitro, the “gold‐standard” assay of tumor cell vaccination was employed.^[^ ^19^ ^]^ Mice were injected with a supposedly preventive vaccine of either argon gas plasma treated or drug‐treated melanoma cells. Seven days later, animals were re‐challenged with live untreated cells, and the number of animals developing tumors was assessed (**Figure** **5**a). While one out of six animals receiving cells treated with the low‐immunogenic drug MMC were protected from tumor growth, it was three out of six for the plasma group and five out of six for the group receiving cells exposed to highly immunogenic drug MTX (Figure 5b). None of the animals developed tumors at the vaccination site. Re‐stimulation of splenocytes isolated from these mice with B16F10 melanoma cells revealed a significant increase of the early activation marker CD69 in CD8^+^ (Figure 5c) but not in CD4^+^ (Figure 5d) T‐cells. To analyze the inflammatory changes associated with the co‐culture of splenocytes or lymph node‐derived cells from the vaccinated animals with melanoma cells ex vivo, 12‐plex bead‐based cytokine and chemokine quantification of the supernatants was performed at 24 h (Figure 5d). Statistical comparison was made by analyzing the *p*‐values of the MMC versus the plasma group and the MTX versus the plasma group. Most significant differences were observed for splenocytes, while for lymph node derived cells, only IL12p70 was significantly elevated in the MTX group. With splenocytes, the plasma group showed significantly enhanced levels of (C‐X‐C motif) ligand 1 (CXCL1), CXCL10, interferon‐gamma (IFNγ), IL1α, IL6, and tumor necrosis factor‐alpha (TNFα) as well as significantly decreased levels of granulocyte‐macrophage colony‐stimulating factor (GM‐CSF) and the chemokine (C‐C motif) ligand 17 (CCL17, also known as TARC) when compared to either MMC, MTX, or both. In total, the chemokine and cytokine expression profile was pro‐inflammatory. It can be concluded that vaccination with plasma‐treated melanoma cells provided immunoprotection from melanoma growth in 50% of mice, and leukocytes from these mice cultured with melanoma cells showed enhanced activation and inflammatory activity that may have supported antitumor immunity in vivo.

## 3 Discussion

Despite improvements in therapy, cancer still ranks first among causes of death in the western world and people younger than 80 years old.^[^ ^20^ ^]^ At the same time, the importance of the immune system to target cancer cells becomes increasingly evident.^[^ ^21^ ^]^ Novel anticancer therapies are hence urgently needed that target cancer cells while fostering antitumor immunity. We investigated a novel anticancer treatment modality, medical gas plasma, in a murine syngeneic model of malignant melanoma and tested its ability to promote antitumor efficacy and immuno‐stimulation.

Gas plasma treatment was effective in inactivating melanoma cells in vitro and reducing tumor mass in vivo. This is in line with previous reports on melanoma using experimental plasma prototypes.^[^ ^9^, ^22^, ^23^ ^]^ However, the translational relevance of these plasma sources is low while it is high for the atmospheric pressure plasma jet kINPen used in this study that is similar to the jet accredited as a medical device in Europe.^[^ ^3^ ^]^ The evidence in the field of medical gas plasma research points to the importance of ROS in mediating plasma‐induced tumor cell death.^[^ ^8^ ^]^ The current concept is that these plasma‐derived ROS generate secondary ROS and oxidation products that accumulate inside tumor cells, leading to mitochondrial damage^[^ ^24^ ^]^ and pro‐apoptotic signaling.^[^ ^25^ ^]^ Exposure of tumor cells to plasma‐derived ROS is accompanied by changes in the release of chemokines and cytokines,^[^ ^26^ ^]^ several immunomodulatory receptors,^[^ ^27^ ^]^ and upregulation of markers of the immunogenic cancer cell death (ICD)^[^ ^28^ ^]^ and cellular senescence.^[^ ^29^ ^]^ While many reports point to a selectivity of gas plasma treatment to induce toxic effects in tumor cells over non‐malignant cells,^[^ ^30^, ^31^, ^32^, ^33^ ^]^ more comprehensive studies revealed that selectivity depended on the type of tumor cell investigated and the cell line used for comparison.^[^ ^34^, ^35^, ^36^, ^37^ ^]^ Notwithstanding, increased expression of pro‐immunogenic surface markers in ROS‐treated cells was observed in malignant over non‐malignant cells.^[^ ^38^ ^]^

With millions of non‐malignant cells dying within the human body each day, apoptosis is per definition an immunologically silent form of cell death. This way, immunological tolerance is maintained toward self‐antigens,^[^ ^39^ ^]^ a mechanism being exploited by tumor cells to evade anticancer immune responses.^[^ ^40^ ^]^ If apoptosis, however, occurs in a highly pro‐inflammatory context, T‐cell co‐stimulation by activated DCs drive antitumor responses critical in the inactivation of cancer cells.^[^ ^41^ ^]^ CRT and heat‐shock proteins are two key immunological determinates in this context,^[^ ^42^, ^43^, ^44^ ^]^ and our results underline their increased surface expression in the immunogenic treatment regimens mitoxantrone (MTX) and gas plasma treatment. This corroborates previous results using another plasma source.^[^ ^9^ ^]^ By contrast, mitomycin C (MMC) induced a tolerogenic form of cell death in both our's and other studies.^[^ ^45^, ^46^, ^47^ ^]^ For this reason, the immunosuppressive capacity of MMC is exploited to reduce graft versus host disease.^[^ ^48^ ^]^ In our study, MMC was the only drug that enhanced the expression of MHC class I molecules on B16F10 cells while other treatment regimens did not. This suggests that antitumor immunity was efficient even at baseline MHCI levels on melanoma cells presenting tumor‐specific peptides. Vice versa, CD47 was markedly increased with gas plasma and—to an even greater extent—MTX treatment. CD47 is a prominent inhibitor of phagocytosis, and its therapeutic blockage has been shown in clinical trials.^[^ ^49^ ^]^ Nonetheless, sufficient immunogenic signaling was shown to overcome CD47‐mediated “don't‐eat‐me” signaling,^[^ ^50^ ^]^ exemplifying the delicate balance of a number of surface molecules determining the efficacy of anticancer responses via immune cells. Along similar lines, our study showed an increased nuclear translocation and hence activation of NFκB, a transcription factor known to promote malignant progression and invasiveness.^[^ ^51^ ^]^ For MTX, however, it is established that such NFκB activation is a consequence of drug‐induced DNA double‐strand breaks and apoptosis.^[^ ^52^ ^]^ With regard to ICD, HSP90 can also inhibit cell death^[^ ^53^ ^]^ through interaction with Akt via NFκB‐mediated apoptosis inhibition.^[^ ^54^ ^]^ In DCs, phosphorylation of NFκB through DAMPs and TLR4 is a critical mechanism of antitumor activity of these cells.^[^ ^55^ ^]^ Necrotic cells, which are pro‐inflammatory and immunogenic per se, can trigger pro‐inflammatory cytokine release through activation of NFκB.^[^ ^56^ ^]^ Gas plasma treatment releases ROS. Another physical clue generating therapeutic ROS is photodynamic therapy, which was shown to stimulate antitumor inflammation via phosphorylation of NFκB.^[^ ^57^ ^]^ Nrf2 is another transcription factor discussed being a tumor promotor and suppressor at the same time.^[^ ^58^ ^]^ Nrf2 translocation to the nucleus was increased with MMC and MTX as well as plasma treatment in tendency and regulates the transcription of antioxidant and anti‐apoptotic genes.^[^ ^59^ ^]^ The increased translocation of Nrf2 was likely due to its redox‐sensitive activation upstream.^[^ ^60^ ^]^ With gas plasma exposure and subsequent ROS deposition onto cells and tissues, Nrf2 activation seems to be a frequently observed process as we recently found its phosphorylation in plasma‐treated wounds in mice.^[^ ^61^ ^]^ Garg and colleagues previously postulated that ICD shares key danger signaling pathways with viral infection,^[^ ^62^ ^]^ in which Nrf2 plays a critical role^[^ ^63^ ^]^ besides its part in the unfolded protein response.^[^ ^64^ ^]^ However, Nrf2 activation can also promote autophagy that counteracts ER stress and ICD.^[^ ^65^ ^]^ As it protects from oxidative stress, it is also thought that excessive phosphorylation of Nrf2 protects cells from dying in an immunogenic manner, even at high cytotoxic dosages of a given ICD inducer.^[^ ^66^ ^]^ In this regard, it is interesting to note that plasma only poorly activated Nrf2 but was highly immunogenic, while MMC and MTX activated Nrf2 to a significantly greater extent. Clearly, the link between oxidative stress and ICD has not been fully elucidated yet. For NFAT, only MTX gave a small but significant increase of nuclear translocation. Hence, its role in ICD, which was clearly elicited in response to gas plasma and MTX treatment, was presumingly little in our model. NFAT activation is involved in TNFα release,^[^ ^67^ ^]^ known to target tumor apoptosis in a T‐cell dependent manner.^[^ ^67^, ^68^ ^]^ Like with the other two transcription factors investigated, however, NFAT was also previously linked to immunosuppressive effects,^[^ ^69^ ^]^ making tissue–environmental factors likely to tip the balance of these pathways toward either tumor promotion or tumor regression.^[^ ^70^ ^]^

Targeting tumor cells with pharmacologically generated ROS has been proposed to be an effective anticancer strategy already a decade ago.^[^ ^71^ ^]^ However, clinical success has so far been limited,^[^ ^72^ ^]^ mainly because of difficulties in targeting therapies in a tumor‐specific way when administered systemically. By contrast, physical methods such as light‐induced photodynamic therapy (PDT),^[^ ^73^ ^]^ ionizing radiation,^[^ ^74^ ^]^ UV‐treatment,^[^ ^75^ ^]^ pulsed‐electric fields,^[^ ^76^ ^]^ and gas plasmas^[^ ^77^ ^]^ generate tumor‐toxic ROS in a localized manner that can moreover then contribute to ICD. However, while the physical modalities mentioned generate ROS in the interior of cells, gas plasma treatment adds ROS from the outside with mechanisms and redox‐chemical reaction pathways only starting to be understood as of now.^[^ ^78^, ^79^, ^80^ ^]^ Due to the mechanism of action, gas plasma produced ROS may only act in a localized manner but not systemically.

The benefit of gas plasma therapy, especially with the jet used in this study, is its multimodal production of a plethora of ROS types simultaneously.^[^ ^81^ ^]^ The two most effective gas plasma settings in our current study, argon and helium/oxygen, were the conditions with most atomic oxygen generation. Hence, we here show for the first time that changing the ROS composition of a gas plasma jet changes the antitumor efficacy against melanoma in a syngeneic animal model. This is a significant step toward the proof‐of‐concept that gas plasma jets can be optimized toward a tumor entity with the potential to serve a novel tool in precision oncology in the future. To identify the ideal gas mixture yielding a maximum antitumor efficacy, however, extensive comparative studies in vivo are needed whose screening nature would not qualify for ethical approval in Germany, at the moment. Not only many more feed gas combinations could be tested (e.g., argon‐oxygen‐nitrogen, argon with nitrogen shielding gas) but also several increments of the additives (e.g., 0.2%, 0.5%, 1%, and 2%). Investigating more iterations will likely optimize antitumor efficacy further, while in this study, we have provided a good starting point suggesting the ·OH‐rich argon gas plasmas and the atomic oxygen‐rich He/O_2_‐gas plasma of a redox‐chemistry having potent tumoricidal effects.

It has also been established with this plasma jet technology that changing the feed gas condition has a significant impact on the ROS composition and its subsequent post‐translational modifications of biomolecules.^[^ ^82^ ^]^ As a functional consequence, some ROS mixtures are associated with a potent cell kill, while others are not.^[^ ^83^ ^]^ We recently identified atomic oxygen, and possibly singlet delta oxygen, to be an essential mediator of toxicity in a leukemia model.^[^ ^84^ ^]^ This was especially evident when oxygen was added to helium, which efficiently generates atomic oxygen at a high concentration as measured before using molecular beam mass spectrometry (MBMS) and two‐photon absorption laser‐induced fluorescence (TALIF).^[^ ^85^ ^]^ Atomic oxygen then is able to generate HOCl in the presence of chloride and liquids, which—at least in tissue cultures—is present at excess. It is crucial to note that this process is highly dependent on the distance of the jet to the liquid as atomic oxygen levels quickly drop with increasing distance from the nozzle.^[^ ^86^ ^]^ Such an effect can also be noticed when analyzing HOCl production as a function of the treatment distance to a target, for example, a liquid.^[^ ^83^ ^]^ This is because ambient air oxygen scavenges atomic oxygen to react to ozone. One question might be why this process is less evident with He/O_2_ while all other conditions (Ar, Ar/O_2_, and He) have higher scavenging rates as seen by the lack of HOCl production in the liquid. First, it needs to be mentioned that the argon and helium settings generate atomic oxygen as well but at concentrations several orders of magnitude lower compared to the respective addition of oxygen.^[^ ^87^, ^88^ ^]^ Second, argon plasma (regardless of addition of 1% O_2_ or not) has high turbulences that lead to intense influx of ambient air into the active plasma zone already at short axial distances from the nozzle.^[^ ^89^ ^]^ Such effect is less pronounced with helium and its lower diffusion coefficient,^[^ ^90^ ^]^ which has a more laminar and not turbulent flow^[^ ^91^ ^]^ as compared to argon. Argon also has a higher diffusion coefficient that exponentially adds to the on‐axis density of the ambient air. This is vice versa suggested by the fact that when the kINPen plasma is shielded with a gas not containing oxygen (e.g., nitrogen or argon), large amounts of atomic oxygen but not ozone are measureable.^[^ ^92^ ^]^ Third, there are many ways of generating atomic oxygen in the complex plasma chemistry that is partly related to molecular gas admixture but also its effects on metastable and electron densities.^[^ ^93^ ^]^

He/O_2_ was the most potent gas mixture for inactivating melanoma cells. In vitro, this might have been due to its high atomic oxygen levels, leading to HOCl production in vitro in the presence of excess liquid and chloride, underlining previous findings.^[^ ^83^, ^84^ ^]^ In a groundbreaking recent study, HOCl was used to prepare autologous tumor material for cell killing and increasing its immunogenicity, which enhanced the antitumor immuno‐protection in patients suffering from ovarian cancer.^[^ ^94^ ^]^ However, also the argon condition was very potent, leading to a significant decline of melanoma growth in vitro and in vivo. The argon plasma is very rich in hydroxyl radical (·OH) generation,^[^ ^95^ ^]^ the most reactive and destructive type of ROS in nature.^[^ ^96^ ^]^ However, ·OH radicals have very short diffusion distances, and quickly deteriorate to H_2_O_2_ in liquids.^[^ ^97^ ^]^ It is vital to note the knowledge gap in redox biology and medicine regarding the spatio‐temporal profiles of different types of ROS, generated via drugs or physico‐chemical means, in tissues. The gas plasma treated tumors in this study were rich in keratins and matrix as well as lipids, with the biomass to liquid ratio being much higher compared to in vitro systems. This means that while laboratory analysis of ROS in the plasma gas phase and liquids might be somewhat accurate, they may not reflect ROS levels in the tissue. As a consequence, the ·OH of the argon gas plasma may promote lipid peroxidation in vivo,^[^ ^98^ ^]^ while in vitro the molecule fails to do so and quickly deteriorates to H_2_O_2_ in the excess liquid. Resolving the trajectories of individual gas plasma‐derived types of ROS in tissues is one of the main technical advances needed at the moment.

Novel treatment modalities require both efficacy and safety. Despite reports with other plasma sources suggesting that antitumor effects of gas plasmas are facilitated via DNA damage,^[^ ^99^, ^100^, ^101^ ^]^ we have no indication of our plasma jet being genotoxic. Our previous studies established that plasma treatment did not cause micronucleus formation in vitro^[^ ^102^ ^]^ regardless of the feed gas settings^[^ ^103^ ^]^ as well as in vivo.^[^ ^104^ ^]^ We also identified that in response to gas plasma exposure, the DNA‐damage indicator γH2AX is a consequence of pro‐apoptotic signaling rather than plasma‐derived ROS directly inducing DNA double‐strand breaks.^[^ ^105^ ^]^ Moreover, a 1‐year follow‐up study in gas plasma‐treated mice confirmed a lack of plasma‐induced tumor formation in vivo.^[^ ^106^ ^]^ Besides the ICD‐inducing nature of plasma‐mediated tumor cell death shown in our present work, gas plasma treatment but not a positive control failed to induce autoimmune events in a previous study in vivo^[^ ^107^ ^]^ that would have occurred in case of an overshooting immune reaction after plasma exposure. In addition, we recently established tumor‐toxic gas plasma treatment^[^ ^108^ ^]^ to be void of pro‐metastatic effects in four human pancreatic cancer cell lines.^[^ ^109^ ^]^

In conclusion, our study suggests medical gas plasma technology to effectively control tumor growth in a syngeneic mouse model of melanoma. Concomitant with enhanced immune cell tumor infiltration and leukocyte activation, we have shown gas plasma treatment to induce immunogenic cancer cell death that protected mice from subsequent tumor growth. Together with previous data on the safety of the medical gas plasma jet system, we propose this technology to be a promising anticancer agent as first reports in patients already suggest. However, the detailed mechanisms of how exactly gas plasma derived ROS penetrate and act on tumor tissue remain to be elucidated in future studies.

## 4. Experimental Section

##### Cell Culture

Highly malignant and metastatic B16F10 murine melanoma cells (ATCC: CRL‐6475) were cultured in Roswell Park Memorial Institute (RPMI) medium containing 10% fetal bovine serum, 2% glutamine, and 1% penicillin/streptomycin (all Sigma). Cells were grown at 37 °C, 95% humidity, and 5% CO_2_, and subcultured twice a week.

##### Medical Gas Plasma Jet Technology

For plasma treatment, an atmospheric pressure argon plasma jet (kINPen) was employed. The device technically is similar to the kINPen MED that has received accreditation as medical device class IIa in Europe and is frequently used in dermatology.^[^ ^3^ ^]^ In standard mode, it is operated using a flow of argon gas (purity 99.9999%; Air Liquide) at three standard liters per minute and a visible plum of about 1 cm. Other feed gas settings were argon plus oxygen, helium, and helium plus oxygen. In the electrode configuration contained within the head of the plasma jet, the noble gas was excited at a frequency of 1 MHz, generating power of about 1 W of the plasma, while total input power was 20 W. For the plasma treatment in vitro, 1 × 10^4^ cells were seeded in 96‐well plates (Eppendorf) having a rim that was filled with double distilled water to minimize edge effects during culture. After adherence overnight, cells were exposed to plasma by guiding the jet's plume over the center of each well for the indicated time in an automated manner. To achieve this, the jet was installed on a *xyz* motorized table (CNC step) controlled via software written to attain sub‐millimeter precision to maximize the reproducibility of the plasma treatment.

##### ROS Detection

For investigating plasma‐derived products, the plasma jet was positioned in front of a UV‐sensitive optical emission spectrometer (Aventes AvaSpec‐2048‐USB2) with a spectral resolution of 0.7 nm and end‐on the plasma jet at a distance of 50 mm from the jet nozzle. The computer‐driven *xyz* motorized table ensured the exact positioning of the plasma jet in this setup. In plasma‐treated liquid, H_2_O_2_ was quantified using the Amplex Ultra Red assay (Thermo) according to the manufacturer's instruction. Fluorescence was determined using a multiplate reader (Tecan F200) at λ_ex_ 560 nm and λ_em_ 590 nm, and absolute concentrations were calculated against a standard curve of H_2_O_2_. Hypochlorous acid was quantified using the taurine chloramine assay. To generate the standard curve, 50 µL of hypochlorite was added to 950 µL of water. 100 µL of this solution was then added to 900 µL of 200 mm KOH (pH 12), and the absorbance was measured at 292 nm using a microplate reader (Tecan M200). The concentration of HOCl was determined using the extinction coefficient of hypochlorite anion. A standard curve was prepared by mixing HOCl with equal volumes of taurine (Sigma) buffer and adding developer solution. The latter consisted of sodium acetate (pH 5.4), sodium iodide, tetramethylbenzidine, and dimethylformamide. The absorbance was measured at 645 nm using a multiplate reader. HOCl concentrations of samples were measured against this standard by adding both taurine buffer and developer.

##### Metabolic Activity and Viability

To analyze the metabolic activity of plasma‐treated B16F10 murine melanoma cells, cells were incubated with resazurin (Alfar Aesar) at a final concentration of 100 µm at 20 h. Resazurin (7‐hydroxy‐3H‐phenoxazin‐3‐one 10‐oxide) is a nontoxic and cell‐permeable dye that is reduced to highly fluorescent resorufin by intracellular enzymes of metabolically active cells. Fluorescence was determined by the utilization of a multiplate reader (Tecan F200) at λ_ex_ 560 nm and λ_em_ 590 nm. Viability was determined microscopically but analyzing terminally dead cells with compromised membranes through which the DNA‐binding dye 4′,6‐diamidino‐2‐phenylindole (DAPI; Sigma) can enter. DAPI was excited at 365 nm using an LED of a fluorescence microscope, and dye‐dependent light emission was captured through a 493 ± 23 nm bandpass filter.

##### Flow Cytometry

Flow cytometry was performed using a 4‐laser (405, 488, 561, and 633 nm) flow cytometer (CytoFLEX S; Beckman‐Coulter) equipped with an autosampler to acquire from 96‐well plates and a three‐laser (405 nm, 488 nm, 638 nm) device (Gallios; Beckman‐Coulter) equipped with an autosampler to acquire from 12 × 75 mm FACS tubes (Sarstedt). Cell suspensions were incubated with a master mix prepared from several monoclonal antibodies conjugated to fluorophores (**Table** **1**). For labeling tumor cell suspensions, antibodies targeting CD3 and labeled with fluorescein isothiocyanate (FITC), F4/80 phycoerythrin (PE), CD62L PE‐dazzle, CD4 PE‐cyanine 7 (PC7), CD11c allophycocyanin (APC), CD45 Alexa Fluor (AF) 700, IAIE APC‐fire, and CD8a brilliant violet (BV) 510 were added together with Sytox Blue (Thermo) to exclude terminally dead cells. After incubation for 30 min on ice, cells were washed twice with running buffer (Miltenyi Biotec) and resuspended in running buffer prior to the acquisition by flow cytometry. To label leukocytes derived from secondary lymphoid organs, the antibody master mix was adjusted to incorporate CD127 FITC and CD69 BV421. Cells of interest were gated from the LIN (lineage)‐negative population labeled with IAIE APC‐fire, CD45R APC‐fire, F4/80 APC‐fire (to gate out myeloid cells, B‐cells, and macrophages), and Zombie (BioLegend) NIR to gate out dead cells in a single dump channel. To assess immuno‐relevant markers on B16F10 melanoma cells, the cells were stained with fluorescently labeled antibodies targeting CD47 PerCP‐Cy5.5, CRT AF647, HSP90 AF700, and MHCI PE. For analysis of viability, B16F10 cells were gas plasma treated and incubated for 24 h at 37 °C. Cells were collected using accutase and washed and stained in annexin V binding buffer (AVBB) containing annexin V‐FITC (both BioLegend) and DAPI (final concentration: 1 µm) for 15 min in the dark. After washing and resuspending in AVBB, the fluorescence per cell was acquired using flow cytometry. Data analysis and display of gating, dot plots, and histograms were performed using Kaluza analysis 2.1.1 software (Beckman‐Coulter). Since a total of more than 250 Mio single cells acquired by flow cytometry were analyzed in this study, high‐performance computing was required using a dedicated Tesla K40 graphics (Nvidia) that utilizes 2880 CUDA cores for parallel computing.

**Table 1:** Antibodies used in this study

Target	Clone	Vendor
CD3	17A2	BioLegend
CD4	L3T4	BioLegend
CD8	53‐6.7	BioLegend
CD11c	HL3	Thermo
CD127	A7R34	BioLegend
CD25	PC61	BioLegend
CD45	30‐F11	BioLegend
CD45R	RA3‐6B2	BioLegend
CD47	miap301	BioLegend
CD62L	MEL‐14	BioLegend
CD69	H1.2F3	BioLegend
CRT	1G6A7	Novus Biologicals
F4/80	BM8	BioLegend
MHCI	28‐14‐8	Invitrogen
HSP90	AC88	Novus Biologicals
IAIE (MHCII)	Sca‐1	BioLegend
NFAT	D43B1	Cell Signaling
NFκB	K10‐895.12.50	BD biosciences
Nrf2	A‐10	Santa Cruz

##### High Content Imaging

A high content/high throughput imaging system (Operetta CLS; PerkinElmer) equipped with a 16‐bit 4.7MP sCMOS camera and a 785 nm laser autofocus was used for quantitative image analysis of transcription factor translocation in B16F10 melanoma cells. After plasma treatment or incubation with either mitomycin C (MMC, final concentration 50 µm) or mitoxantrone (MTX, final concentration 50 µm), cells were fixed and permeabilized, and stained with antibodies for 1 h at 37 °C. DAPI was used as a counterstain for nuclei. 96‐well glass‐bottom plates (PerkinElmer) were used to facilitate the use of a 20× water immersion objective (NA 1.0; Zeiss) for maximum photon counts on the photomultiplier. Excitation and emission settings were λ_ex_ 475 nm and λ_em_ 525 ± 25 for AF488, λ_ex_ 550 nm and λ_em_ 610 ± 40 for AF594, and λ_ex_ 630 nm and λ_em_ 708 ± 52 for AF647, respectively. For each condition, about 50 000 individual cells were analyzed using algorithm‐driven quantitative image analysis facilitated using Harmony 4.9 software (PerkinElmer). The analysis sequenced included segmentation of nuclei via DAPI and finding of the cytosolic region of each cell using the digital phase contrast (DPC) channel of the system in a label‐free manner. Subsequently, the mean fluorescence intensity (MFI) of each transcription factor in the nucleus was calculated over that of the cytosol and given as N/C ratio.

##### In Vivo Anti‐Melanoma Plasma Jet Therapy

The ethical implications of the experiment were reviewed and approved by the local authority *Landesamt für Landwirtschaft, Lebensmittelsicherheit und Fischerei* (LALLF) *Mecklenburg‐Vorpommern* (approval number M‐V 7221.3‐1‐023/17). Wildtype C57BL/6 mice were shaved on the flank and inoculated with 1 × 10^5^ syngeneic murine B16F10 melanoma cells in 50 µL of phosphate‐buffered saline (PBS). For plasma treatment, the tumors were exposed to the gas plasma for 4 min during each intervention cycle. Feed gas settings were argon, argon plus 1% oxygen helium, and helium + 1% oxygen. As a positive control, imiquimod (Aldara) was applied via creaming the inoculation area. This small molecule is used to treat human metastatic melanoma in the skin of patients,^[^ ^110^, ^111^, ^112^ ^]^ and its clinical relevance makes this drug an excellent positive control. Its mechanism of action is that it acts as toll‐like‐receptor 7 (TLR7) agonist, which leads to the recruitment of myeloid cells, such as dendritic cells, into the tumor microenvironment (TME),^[^ ^113^ ^]^ which potentiates antitumor immunity. The second mode of action is its potent inhibition of complex I in the mitochondrial membrane, which is being discussed as an additional antitumor mechanism.^[^ ^114^ ^]^ In another animal group in our experiments, imiquimod was added, followed by argon plasma treatment. This combination was chosen because both imiquimod and the argon‐driven plasma jet are accredited clinical procedures already. Compelling evidence of our and future studies may, therefore, motivate an investigator‐initiated clinical trial. Feed gas combinations other than argon alone may need accreditation according to the medical device regulation in Europe first before its clinical use could be envisaged. In the control group, tumors were left untreated. After sacrifice, tumors and secondary lymphoid organs (spleens, lymph nodes) were explanted. Tumors were weighed. Single‐cell suspensions of tumors were retrieved using the GentleMacs tumor dissociation kit mouse (Miltenyi) and the OctaMacs device (Miltenyi) and subjected to analysis by flow cytometry. Viable cell suspensions of spleens and lymph nodes were retrieved using the spleen dissociation kit (Miltenyi) and the OctaMacs device, prior to the flow cytometric analysis. In addition, 1 × 10^6^ splenocytes were cultured for 18 h in the presence or absence of 1 × 10^5^ heat‐inactivated (3 min, 65 °C) B16F10 cells in 24‐well plates (Eppendorf), and investigated by flow cytometry thereafter.

##### In Vivo Anti‐Melanoma Vaccination

The ethical implications of the experiment were reviewed and approved by the local authority *Landesamt für Landwirtschaft, Lebensmittelsicherheit und Fischerei* (LALLF) *Mecklenburg‐Vorpommern* (approval number M‐V 7221.3‐1‐023/17). Seven‐hundred thousand melanoma cells were exposed to argon plasma or drugs. The latter were either the positive control MTX (final concentration 10 µm) or the negative control MMC (final concentration 50 µm). The cells were cultured in a flask for 24 h, before dislodgement using accutase (BioLegend), and resuspension in 700 µL of PBS; 100 µL of this suspension was injected into the left flank (vaccination) of wildtype C57BL/6 mice (six mice per group). Seven days later, 1 × 10^5^ syngeneic murine B16F10 melanoma cells in 50 µL of PBS were inoculated in the right flank of the animals (re‐challenge). On the day of sacrifice, tumor growth was inspected at the re‐challenge injection site. Secondary lymphoid organs were harvested, and single‐cell suspensions were retrieved as described above. Cell suspensions were cultured in the presence of B16F10 melanoma cells for 18 h. Flow cytometric analysis of splenocytes was performed. In addition, supernatants were collected and stored at −20 °C prior to analysis by multiplex bead based quantification of chemokines and cytokines (LEGENDPlex; BioLegend). Quantification of the 12 analytes was performed according to the manufacturer's instructions and analyzed using the LEGENDplex data analysis software utilizing an *R*‐package.

##### Statistical Analysis

Graphing and statistical analysis were performed using prism 8.3 (Graphpad software). Comparison of two groups was made using unpaired student's *t*‐test. The comparison of more than two groups was made using a one‐way analysis of variances (ANOVA). The comparison of more than two groups across different immune cell subpopulations was made using two‐way ANOVA. Level of significance is indicated as follows: α = 0.05 (*), α = 0.01 (**), α = 0.001 (***).

## Conflict of Interest

The authors declare no conflict of interest.

## Author Contributions

E.F. and A.S. contributed equally to this work. S.B. designed the study, analyzed the data, and wrote the manuscript. R.C., F.N., S.K.S., E.F., and A.S. performed the experiments, analyzed the data, and reviewed the manuscript.
